# An optimized algorithm for detecting and annotating regional differential methylation

**DOI:** 10.1186/1471-2105-14-S5-S10

**Published:** 2013-04-10

**Authors:** Sheng Li, Francine E Garrett-Bakelman, Altuna Akalin, Paul Zumbo, Ross Levine, Bik L To, Ian D Lewis, Anna L Brown, Richard J D'Andrea, Ari Melnick, Christopher E Mason

**Affiliations:** 1 Department of Physiology and Biophysics, 1305 York Ave., Weill Cornell Medical College, New York, NY 10065, USA; 2 The HRH Prince Alwaleed Bin Talal Bin Abdulaziz Alsaud Institute for Computational Biomedicine, 1305 York Ave., Weill Cornell Medical College, New York, NY 10065, USA; 3 Department of Medicine, Division of Hematology/Oncology, 1300 York Ave., Weill Cornell Medical College, New York, NY 10065, USA; 4 Human Oncology and Pathogenesis Program, Memorial Sloan-Kettering Cancer Center, 1275 York Avenue, Box 20, New York, NY 10065, USA; 5 Directorate of Haematology, SA Pathology and Department of Haematology, Royal Adelaide Hospital, Adelaide, South Australia; 6 Directorate of Haematology and Centre for Cancer Biology SA Pathology, The Queen Elizabeth Hospital, Woodville, South Australia; 7 Department of Haematology and Oncology, The Queen Elizabeth Hospital, Woodville, South Australia; 8 Department of Pharmacology, 1300 York Ave., Weill Cornell Medical College, New York, NY 10065, USA

## Abstract

**Background:**

DNA methylation profiling reveals important differentially methylated regions (DMRs) of the genome that are altered during development or that are perturbed by disease. To date, few programs exist for regional analysis of enriched or whole-genome bisulfate conversion sequencing data, even though such data are increasingly common. Here, we describe an open-source, optimized method for determining empirically based DMRs (eDMR) from high-throughput sequence data that is applicable to enriched whole-genome methylation profiling datasets, as well as other globally enriched epigenetic modification data.

**Results:**

Here we show that our bimodal distribution model and weighted cost function for optimized regional methylation analysis provides accurate boundaries of regions harboring significant epigenetic modifications. Our algorithm takes the spatial distribution of CpGs into account for the enrichment assay, allowing for optimization of the definition of empirical regions for differential methylation. Combined with the dependent adjustment for regional p-value combination and DMR annotation, we provide a method that may be applied to a variety of datasets for rapid DMR analysis. Our method classifies both the directionality of DMRs and their genome-wide distribution, and we have observed that shows clinical relevance through correct stratification of two Acute Myeloid Leukemia (AML) tumor sub-types.

**Conclusions:**

Our weighted optimization algorithm eDMR for calling DMRs extends an established DMR R pipeline (methylKit) and provides a needed resource in epigenomics. Our method enables an accurate and scalable way of finding DMRs in high-throughput methylation sequencing experiments. eDMR is available for download at http://code.google.com/p/edmr/.

## Background

Advanced, high-throughput sequencing technologies allow for fast, single-base resolution scans of entire epigenome. Large-scale sequencing projects are producing these datasets for cancer research, and these epigenetic marks provide important information about cellular phenotypes in normal and diseased tissues [1,2]. DNA methylation pattern changes are pivotal marks needed in cells' differentiation during tissue and lineage specification, and, as such, contribute to the complexity of organisms' cellular sub-types [3,4]. Furthermore, aberrant DNA methylation not only defines malignant subtypes of disease [5,6], but also contributes to malignant disease pathophysiology and can be used in clinical outcome predictions [7].

Bisulfite sequencing of genomic DNA is a widely applied method for methylation measurement. Whole-genome bisulfite sequencing is a genome-wide technique for the measurement of DNA methylation [8]. However, other enrichment DNA methylation sequencing methods have been developed to achieve cost-effective coverage of variable regions of DNA methylation. These methods often utilize reduced representation of bisulfite sequencing by focusing on restriction sites, including methods such as Reduced Representation Bisulfite sequencing (RRBS) [9-11], Enhanced RRBS (ERRBS) [12], multiplexed RRBS [13], methylation-sensitive restriction enzyme sequencing [14], as well as other enrichment approaches, including methylated DNA immunoprecipitation sequencing [15] and methylated DNA binding domain sequencing [16].

Previously, epigenome analysis tools such as methylKit [17] have focused on comprehensive DNA methylation analysis of single base sites, in order to find differentially methylated cytosines (DMCs). However, biological regulation by methylation can be mediated by a single CpG or by a group of CpGs in close proximity to each other. Therefore, a combination of baseresolution analysis and regional analysis of DNA methylation may offer a more comprehensive and systematic view of bisulfate sequencing data. This increasing demand for tools to find differentially methylated regions (DMRs) increases as more data emerge from both large-scale epigenomics consortiums and from individual labs. To address this need, we have created eDMR, which exists as stand-alone code for use with other tools and packages. It can also be used as an expansion of the methylKit R package for comprehensive DMR analysis. eDMR can directly take objects from methylKit or data frames with differential methylation information, or any DMC result in bed file format, and perform regional optimization calling and DMR statistical analysis and filtering. Furthermore, eDMR offers annotation functions that map DMRs to gene body features (coding sequences, introns, promoters, 5' untranslated regions (UTR), and 3'UTR), CpG island and shore locations, as well as the use of any other user-supplied coordinate files for annotation. Here, we describe an example of using eDMR with DNA methylation data from the ERRBS protocol.

## Methods

### Data source

Ten acute myeloid leukemia (AML) de-identified patient samples enriched for myeloblast cells and five normal bone marrow (NBM) samples (purchased from AllCells) were used in the experiments. Institutional review board approval was obtained at Weill Cornell Medical Center and at the Royal Adelaide Hospital and this study was performed in accordance with the Helsinki protocols. DNA was extracted using standard techniques and ERRBS library preparations were performed as previously described [12]. Libraries were sequenced on a HiSeq2000 Illumina machine using 75 bp single-end reads to an average depth of 79X per covered CpG. A previously published dataset of two AML subtypes (IDH mutants and MLL rearranged) and two CD34+ normal bone marrow controls [12] (GEO accession number GSE37454) was also used in the analysis.

### Computational tools

R version 2.15.2 [18] and methylKit 0.5.6 [17] were used for the analysis. eDMR depends on Bioconductor packages [19], including methylKit 0.5.6 [17], GenomicRanges 1.8.13, data.table 1.8.6, mixtools [20], doMC 1.2.5, ggplot2 0.9.3 [21].

### Data preprocessing

We performed bisulfite treated read alignment to hg19 genome and methylation calls as previously described [12]. Five NBM samples served as controls for the AML samples. The total coverage for each CpG in the controls is the sum coverage from 5 NBM samples. The methylation level for each CpG in the control is the mean of all NBM samples. We required the coverage of each CpG site be equal or greater than 10X in at least 3 NBM samples for control. For AML samples, the coverage for each CpG site for each sample is 10X. We used fisher exact test from methylKit [17] to compare AML samples with control.

### eDMR algorithm

**Figure 1:**
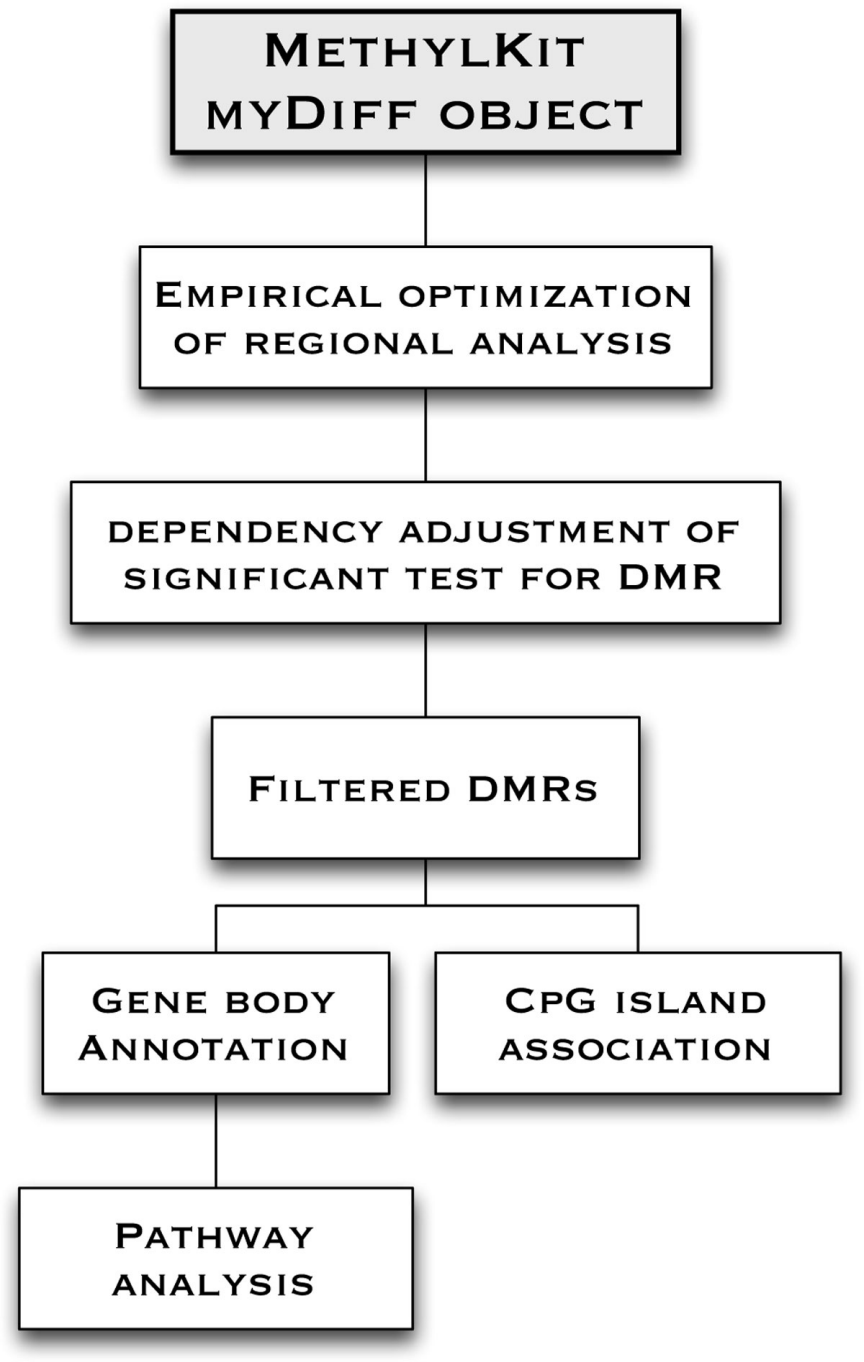
**Workflow of DMR analysis**. Objects and data frames from the R-package methylKit (top, grey), or other DNA methylation base-pair data outputs, can immediately be utilized in all the functions in eDMR (white, below).

Our eDMR algorithm contains five distinct components, which are described below (Also see Figure 1 for a workflow of the eDMR analysis). Our definition of a DNA methylated region is a cluster of CpGs in close spatial proximity. If two adjacent CpGs are separated by more than a certain (algorithm-specified) genomic distance, we define them as coming from different methylated regions. If two CpGs are within a specified genomic distance from each other, then we define them to be within the same region. The eDMR algorithm aims to optimize the threshold for determining DNA methylation regions and to perform statistical significance testing.

#### 1. Empirical regions boundary determination

We used a bimodal normal distribution to identify the optimum cutoff for calling a gap between two DMRs. First, we examined the distribution of the distance between adjacent CpGs (with a coverage >= 10X) across the genome from Sample 1 of our AML dataset. After a log2 transformation, we observed a bimodal distribution with a spike at log2 distance = 0 (Additional file 1: Figure S1). This spike represents the reverse complement of CpGs (GpCs) on the other strand, which has a distance of 1 bp (log2 (1) = 0). Disregarding the strand of CpGs, the base pair distance is counted as 1 bp (log2 (1) = 0). Because we expect the threshold of gap between adjacent methylated regions will be much greater than 1 bp, the frequency for this portion does not contribute to our decision process for DMR determination. After removing the first spike at 0 log2 distance, we then used this dataset with the application of the expectation maximization (EM) algorithm to fit to a bimodal normal distribution [20].

$$F\left(x\right)=\overset{2}{\underset{i=1}{\sum }}\lambda_{i}P_{i}\left(X\leq x\right)$$

Where x is the log2 distance of two nearest CpGs, F is the probability density function (P.D.F) for the mixed normal distribution to which we are trying to fit, and we have *i *= {1,2} as the first/second normal distribution from the bimodal distribution for regional/boundary CpGs. Here $\sum_{i=1}^{2}\lambda_{i}=1$, stands for the two mixing proportions of the two populations.

We then sought to determine the best separation point between the two normal distributions, which will help determine the cutoff of log2 distances between the nearest two CpGs at DMR boundaries. Since the distributions overlap in ERRBS data, we risked mislabeling components from one population to another. However, ERRBS is an enrichment assay, and, as such, the detected CpGs were not evenly distributed along the genome. Instead, the CpGs formed in clusters. This contributed to the imbalance of the two populations (regional CpGs' distance distribution and boundary CpGs' distance distribution). To account for this difference, we used a weighted, combined probability function C(x) to evaluate and characterize the cumulative cost of any given separation point *x*.

$$C\left(x\right)\;=\;\lambda_{1}P_{1}\left(X\geq x\right)\;+\;\lambda_{2}P_{2}\left(X\leq x\right)$$

**Figure 2:**
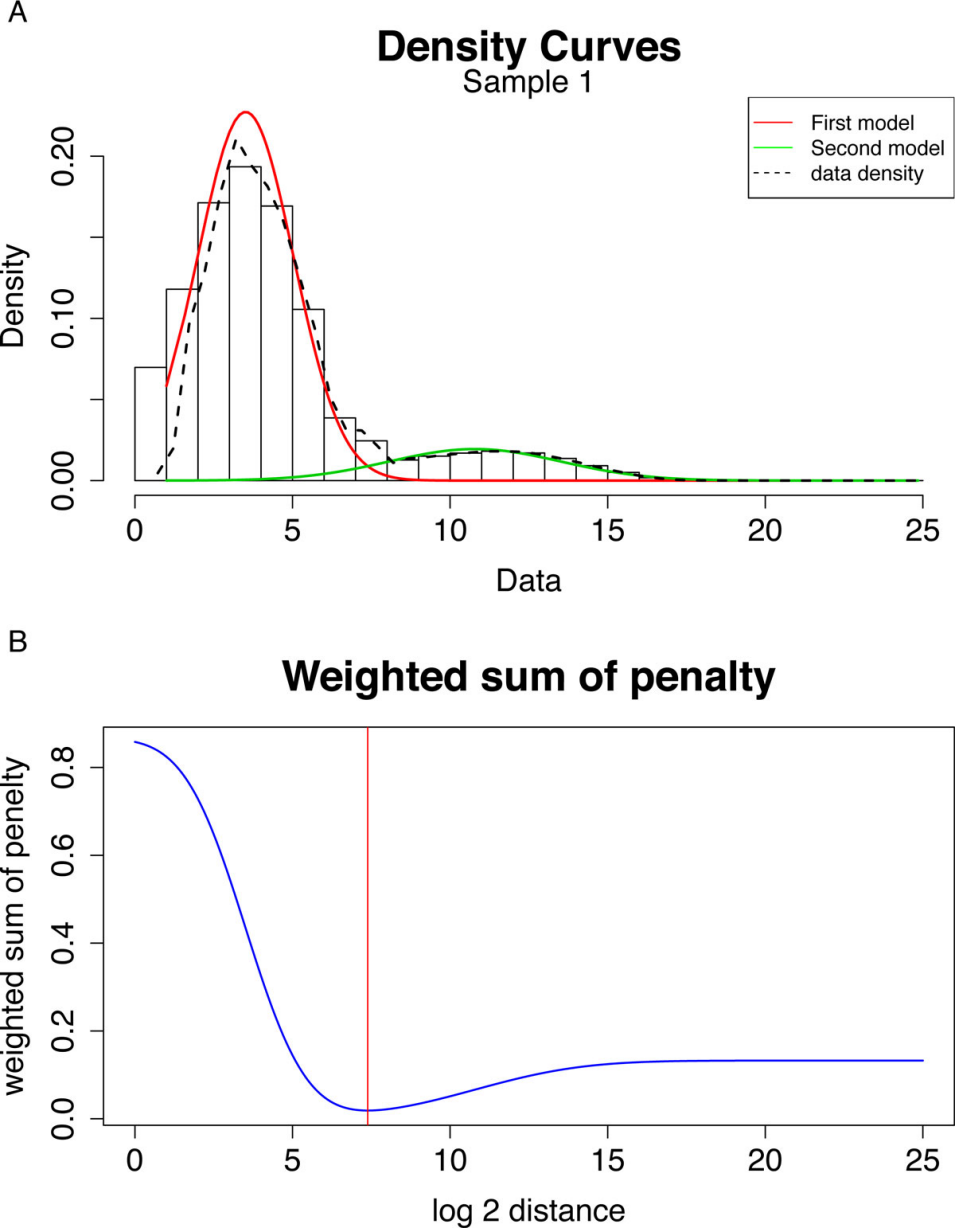
**Identification of the optimal cutoff for calling a gap between two DMRs**. (A) Histogram of the log2 distance of the nearest CpGs in Sample 1. A spike at zero log2 base pairs distance represents the reverse complement of CpGs (GpC) on the other strand. (B) Bimodal normal distribution fitting on the log2 distance of adjacent CpGs genome-wide in AML sample 1. Two distributions (red, and green) are shown that account for two separate data densities (dotted line). (C) Weighted sum of penalty changes (blue line) over log2 distances. The red line is the optimized log2 DMR distance with the lowest weighted penalty from the cost function

In order to minimize the error rate from both populations, we used this weighted combined probability function C(x) to evaluate and optimize the separation of the two populations (Figure 2B). Because the majority of the distances fell into the first normal distribution for regional CpGs (Figure 2A), the weighted model imposed a greater penalty for the probability of mislabeling the CpGs from the first distribution, thus ensuring that we evenly penalized the number of mislabeled CpGs from both distributions, using:

$$\hat{x}\;=\;argmin_{x}\left\{\lambda_{1}P_{1}\left(X\geq x\right)\;+\;\lambda_{2}P_{2}\left(X\leq x\right)\right\}$$

Where P_1 _is the fitted P.D.F. of the first normal distribution for regional CpGs, and P^2 ^is the fitted P.D.F. of the second normal distribution for boundary CpGs, where $\sum_{i=1}^{2}\lambda_{i}=1$, stands for the mixing proportions for the two populations. We then used the successive parabolic interpolation from R stats package to search the interval from the lowest log2 distance to the maximum log2 distance to obtain the minimum of the weighted sum of the cost function C (red line in Figure 2B). The corresponding log2 distance was used for raw region determination.

#### 2. Empirical regions filtering and characterization

Once the regional boundaries for CpG distances (*D) *were determined from the cost function, we examined all distances of the nearest CpGs along the same chromosome. If any distance was greater than *D*, then we called the two CpGs associated with this distance as the boundaries of two regions. We then further refined our distance based on the following independent (and adjustable) filters to increase the power of DMR detection:

1) At least 1 DMC in the region, as determined using, for example, methylKit [17]

2) At least 3 CpGs included in the region, and

3) Absolute mean methylation difference greater than 20%.

#### 3. Statistical significance calculation for DMRs

In order to perform a dependency adjusted significance test, we first examined the spatial auto correlation of methylation data. We created an adjustable spatial parameter (default = 100 base pairs) that binned the data into segments and then calculated the auto correlation based on both the methylation changes and the p-values for each bin [22]. Based on the refined regions, we calculated the significance of the regions by combining the p-values of DMCs within that region. We used the dependence adjustment of the Stouffer-Liptak test to combine p-values [22,23]. Unlike the adjustment for the Fisher's combined probability test, the Stouffer-Liptak joint p-value does not depend on the assumption that the p-values are normally distributed, and thus can be applied for nonparametric data. A FDR (False Discovery Rate) correction was also applied to correct for multiple hypothesis testing for the combined p-values [24,25].

#### 4. Whole methylome DMR characterization: descriptive statistics and sample clustering

While raw output from filtered DMRs are useful, algorithms that contextualize and categorize changes from genomics assays help subsequent analysis. To aid in such global examinations, we also provide convenient functions to examine the DMRs for a given dataset, including data about the distribution of the length of DMRs, overall methylation difference distribution, and the number of DMCs in each DMR for all the samples. These tools give users an easy means to examine broad questions about genome biology and DMR localization for a given set of samples, or to find outlier samples from experimental datasets.

#### 5. DMR annotation with gene models and CpG island

Lastly, we provide a comprehensive gene annotation set which can be used with the coordinates of the DMRs to provide information about gene models, but also characterize DMR changes for different parts of the gene body, separated into: coding sequence, introns, promoters, 5' UTR and 3' UTR. We also allow users to compare to CpG islands, shores, and other user supplied epigenetic loci, such as ENCODE enhancers.

## Results and discussion

### eDMR definition

To determine DMRs, we sought to determine the optimal parameters for regional analysis. ERRBS and other enriched bisulfate conversion sequencing techniques are designed to cover cytosines in CpG-enriched regions, such as CpG islands and regions surrounding digestion sites from restriction enzymes. To accurately define the distinct genomic regions of DNA methylation, we first examined the distribution of the genomic distance between adjacent CpGs covered in the ERRBS data (Additional file 1: Figure S1). Since ERRBS uses the MspI restriction enzyme to fragment DNA, we detected CpGs clustered in CpG-rich regions, which were in close proximity to each other.

These CpG distances established a range of CpGs in close spatial proximity, and the distance cutoff was next determined by eDMR. If two CpGs were far away from each other, then we defined them as coming from different methylated regions (boundary CpGs); on the other hand, if two CpGs were close to each other, then we defined them as coming from the same region (regional CpGs). The eDMR algorithm optimized the threshold for calling methylated regions and performed statistical tests on the methylated regions. A nonparametric density plot of the distribution of the log2 distance of the nearest CpGs showed compelling evidence for a bimodal distribution (Figure 2A and Additional file 1: Figure S1, dashed line). We assumed that the first mode was composed of regional CpGs and that the second mode was composed of boundary CpGs, for the following reasons: (1) the mean of the first mode was less than the mean of the second mode, and (2) the first mode of the bimodal distribution had a larger mixing proportion than the second mode (Figure 2A).

To determine the optimum cutoff of two adjacent CpGs for calling a DMR boundary, we then sought to determine the best separation point between the two normal distributions. We used this weighted combined probability function C(x) to evaluate and optimize the separation of the two populations (Figure 2B). We then determined the minimum of the weighted sum of the cost function at the red line in Figure 2B.

This information was used in our eDMR algorithm to identify the optimum cutoff for calling a gap between two DMRs. This approach was tested on an additional set of 14 ERRBS samples (9 acute myeloid leukemia (AML) and 5 normal bone marrow controls), which revealed similar bimodal distributions (Additional file 1: Figure S2). The mean optimum distance cutoff for all 10 comparisons is 183.50 with standard error of the mean 5.08 (183.50 ± 5.08). After determining the statistically significant DMRs between two samples or groups, the regions were filtered further based on the number of DMCs (minimum of one) and CpGs (minimum of three) within the area, as well as the mean methylation difference (greater than 20%). eDMR can utilize data from methylKit and other DNA methylation pipeline outputs for analysis as well as usersupplied coordinate files for annotation (See Figure 1 for a workflow of the eDMR analysis).

### eDMRs can accurately discern leukemia tumor sub-types

**Figure 3:**
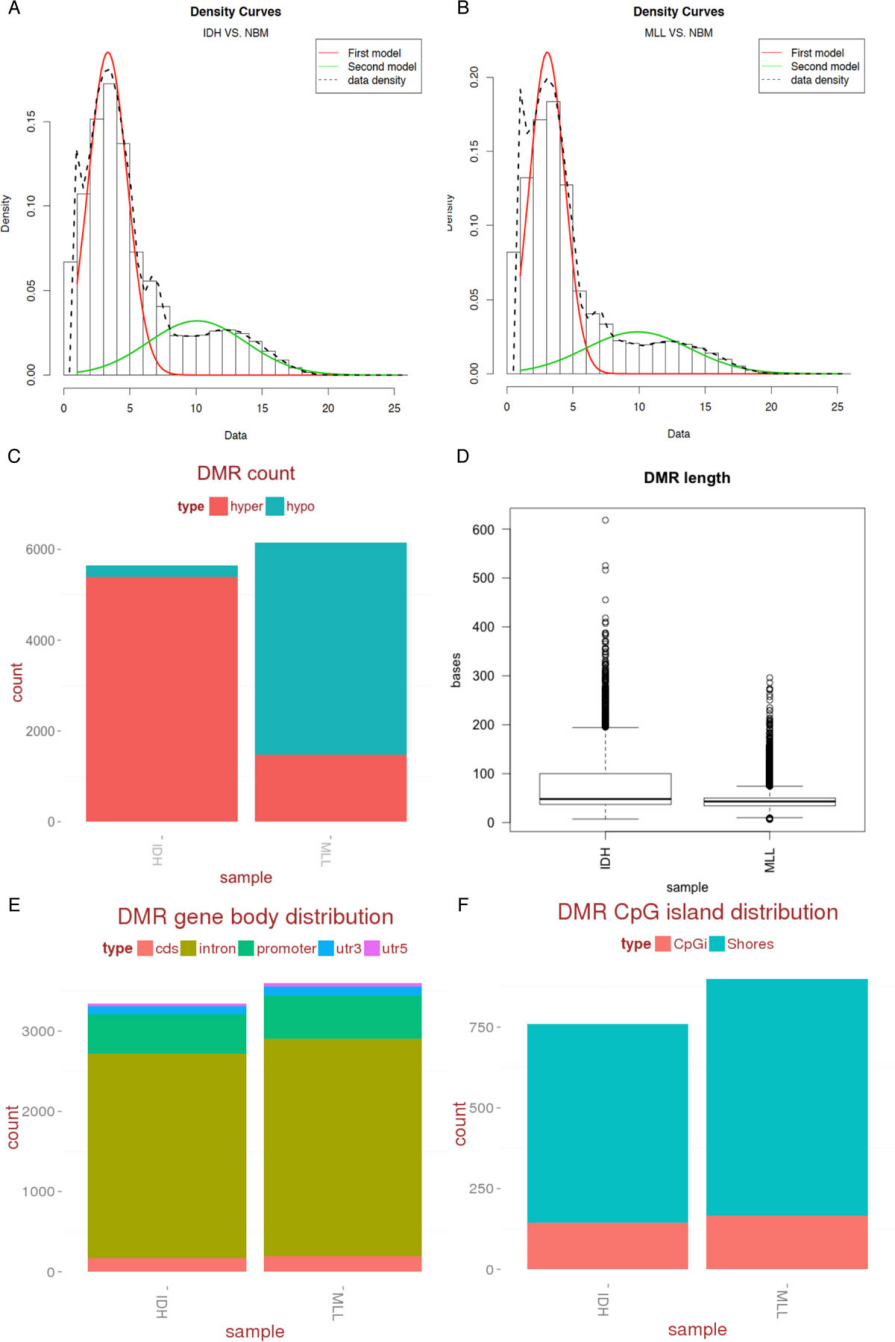
**DMR analysis and output of eDMR for leukemia samples**. (A) Fitting of the bimodal normal distribution to CpGs common to the IDH AML and normal bone marrow control samples. (B) Fitting of the bimodal normal distribution to CpGs common to the MLL AML and normal bone marrow control samples. Both data have similar distributions. (C) The number of hypermethylated (red) and hypomethylated (blue) DMRs identified in each leukemia subtype. (D) Boxplots of the DMR length distributions in both leukemia subtypes. (E) Gene body distributions for CDS (red), introns (mustard), promoters (green), 3'UTRs (blue), and 5'UTRs (purple). (F) CpG island (red) and shore (blue) DMR count distribution in the IDH and MLL AML tumor-types.

We next used a set of previously published leukemia ERRBS data [12] that demonstrated distinct epigenetic tumor sub-types when examined at the level of DMCs. The CpG genomic distribution in these samples also had a bimodal distribution (Figure 3A and 3B). We used eDMR to calculate the number of DMRs between the two tumor sub-types (IDH and MLL) and the normal controls. Similar to previous findings using DMCs alone, unique patterns of DMRs were detected in the two leukemia sample subtypes (Figure 3C). Specifically, IDH AMLs had more hypermethylated DMRs while MLL AMLs had more hypomethylated DMRs. Notably, eDMR revealed that the two tumor sub-types also showed differing DMR lengths (Figure 3D; p = 2.2 × 10^-16^, Kolmogorov-Smirnov test), which showed that our method can replicate previous results and also provide further insight into the epigenomic landscape of these two AML subtypes.

We then examined the spatial changes of the DMRs relative to other genome features. It has been reported that DNA methylation of different parts of the gene body may exert alternate effects on gene expression. Indeed, methylation on promoter regions of the gene tends to have inverse association with gene expression, while genic methylation changes have a more positive correlation [26]. Thus, we sought to curate DMRs with a detailed annotation map, and eDMR functions were created to accomplish this task. We annotated the DMRs identified in the AML samples using Refseq gene models, separated into coding sequence, introns, promoters, 5' UTR and 3' UTR (Figure 3E). We also annotated DMRs with CpG islands and shores (Figure 3F). These separate gene and genomic geographies allow a more granular examination of the underlying methylation changes in a dataset that may have a regulatory impact on gene transcription.

## Conclusion

Profiling DNA methylation changes is a broadly studied topic for basic research across many laboratories. These data are being generated in several large-scale projects, including the Encyclopedia of DNA Elements (ENCODE) Consortium (http://genome.ucsc.edu/ENCODE/), Epigenomics RoadMap (http://www.roadmapepigenomics.org/), and the EU's Blueprint Project (http://www.blueprint-epigenome.eu/). All of these projects provide an abundance of DNA methylation and epigenetic data using DNA methylation sequencing methods like ERRBS, as well as other per-base epigenetic information. Having the ability to dissect the patterns of DNA methylation changes from a regional perspective, rather than at a per-base level, is important for researchers to more completely understand the effects DNA methylation changes have in normal and diseased samples.

Here, we described eDMR - a set of convenient tools for regional analysis of methylation with optimization algorithms. These independent tools can also be utilized in concert with an existing, open-source R-package that automates other aspects of ERRBS analysis (methylKit) such as data processing and DMC analysis. As such, these methods are suitable for any base-level dataset of reduced representation or other base-level DNA modification data sets. These methods are efficient with existing datasets, recapitulate the characterized tumor-subtypes from a positive control data set, and find new aspects of the tumor biology that can only be discovered using a regional analysis. Also, we note that we have used these tools on 15 samples and found the methods to be robust on ERRBS data from both different sample types and at a variety of sequencing depths. All together, these results support the utility of eDMR as a broadly relevant method for DMR characterization, which can be used to further discoveries of epigenetic and regulatory changes and help discern the relevance of DMRs to disease biology in conjunction with other molecular profiling data types.

## List of abbreviations used

AML: Acute Myeloid Leukemia; DMC: differentially methylated cytosine; DMR: differentially methylated region; eDMR: empirically-based differentially methylated regions; ERRBS: Enhanced Reduced Representation Bisulfite sequencing; FDR: False Discovery Rate; RRBS: Reduced Representation Bisulfite sequencing; P.D.F: probability density function; UTR: Untranslated region.

## Competing interests

The authors declare that they have no competing interests.

## Authors' contributions

SL designed the software, processed the data, and performed the analysis. CEM and SL conceived of the algorithm and wrote the manuscript. AA, CEM, FEG-B, and PZ participated in the design of the software. FEG-B performed the RRBS experiments. AM, CEM, FEG-B, RL, and SL contributed to the patient data sets and analysis ideas. AB, BK, LL, and RD contributed materials. All authors critically reviewed and edited the manuscript prior to submission.

## Supplementary Material

Additional file 1 **Figure S1**. Histogram of the log2 distance of the nearest CpGs in Sample 1. A spike at zero log2 base pairs distance represents the reverse complement of CpGs (GpC) on the other strand.
**Figure S2**. Consistent distribution shapes across samples. Samples 2-10 are shown from different sequencing depths and samples. (A-I) Red line: First model for regional CpGs; green line: fitted second model for boundary CpGs; Dashed line: density plot of the log2 distances of the nearest CpGs. Click here for file

## Declarations

Publication of this article was supported by NIH grants R01HG006798, R01NS076465, and R44HG005297. FEG-B was supported by Sass Foundation Judah Folkman Fellowship and NIH grant K08CA169055.

This article has been published as part of *BMC Bioinformatics *Volume 14 Supplement 5, 2013: Proceedings of the Third Annual RECOMB Satellite Workshop on Massively Parallel Sequencing (RECOMB-seq 2013). The full contents of the supplement are available online at http://www.biomedcentral.com/bmcbioinformatics/supplements/14/S5.
