# Supplementary figures and images for: An optimized algorithm for detecting and annotating regional differential methylation
Source: BMC Bioinformatics. 2013 Apr 10;14(Suppl 5):S10. doi: 10.1186/1471-2105-14-S5-S10 (PMC3622633; doi:10.1186/1471-2105-14-S5-S10)

**Figure S1**

**Histogram of log 2 distance of nearest CpGs**

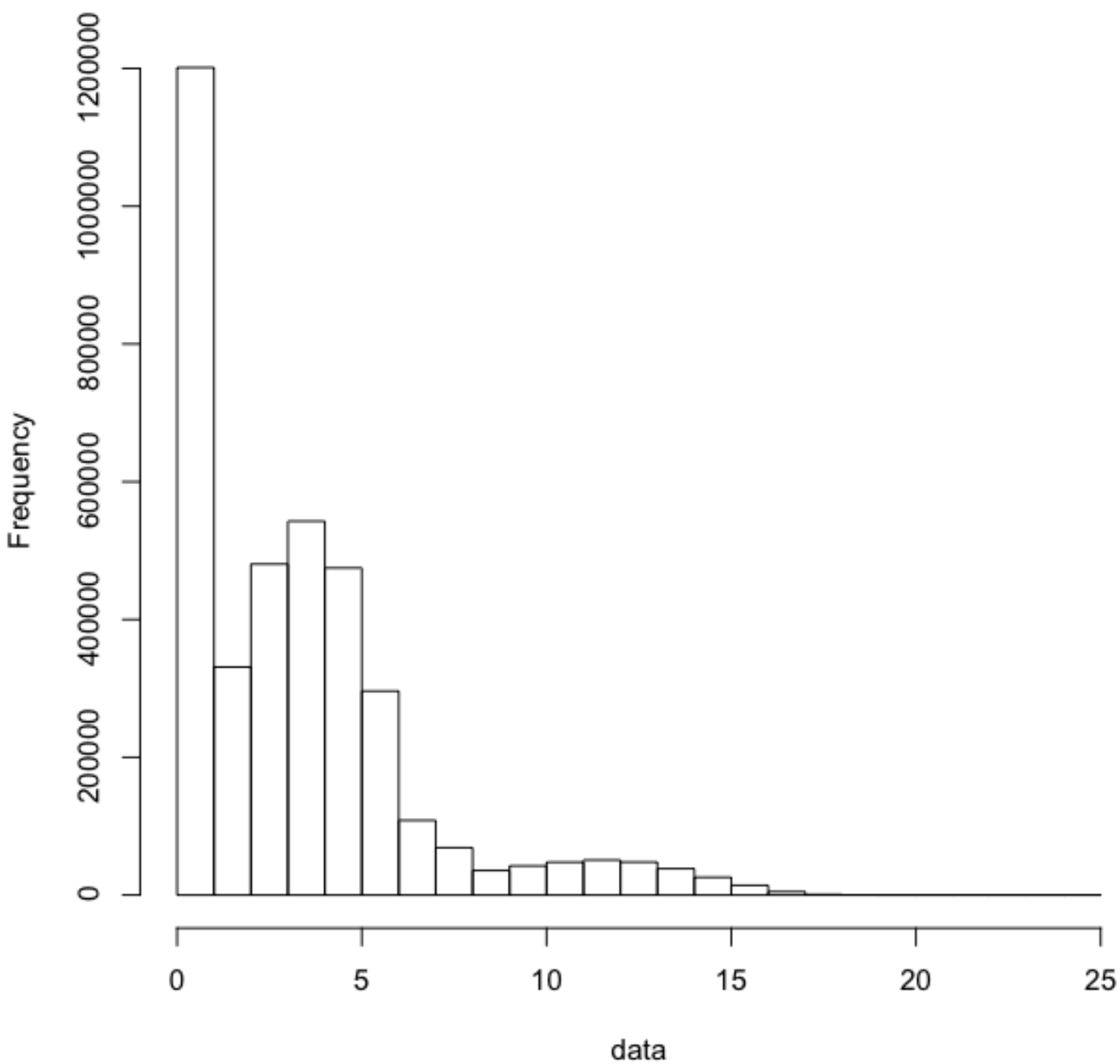

A

A

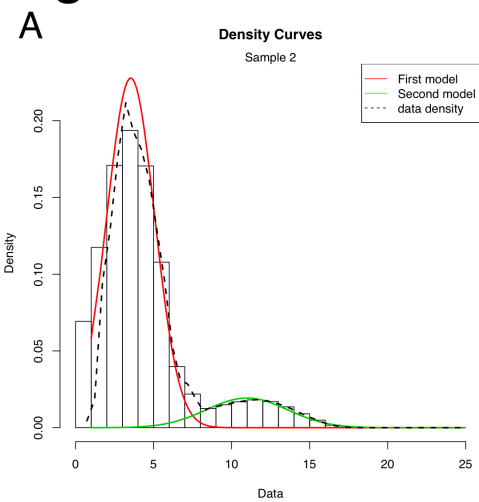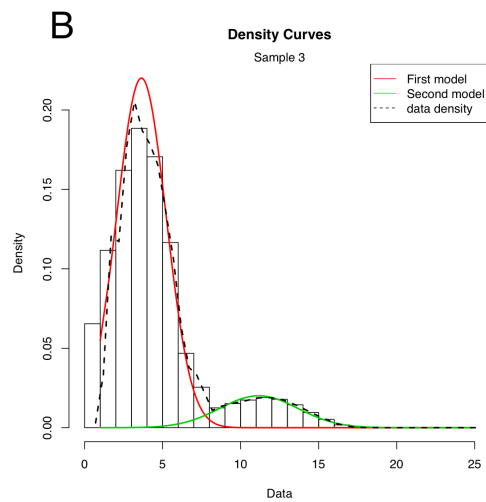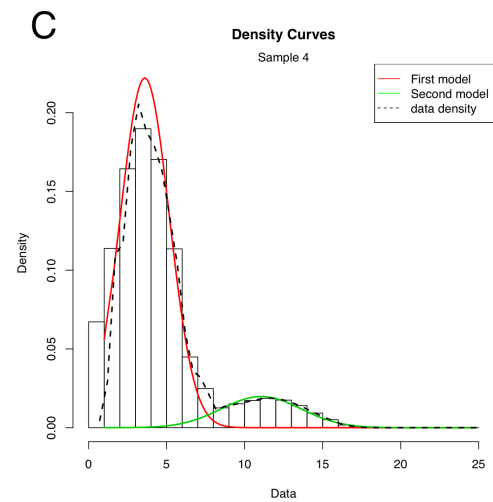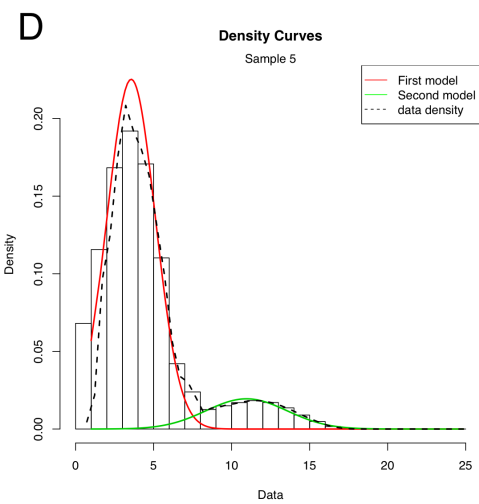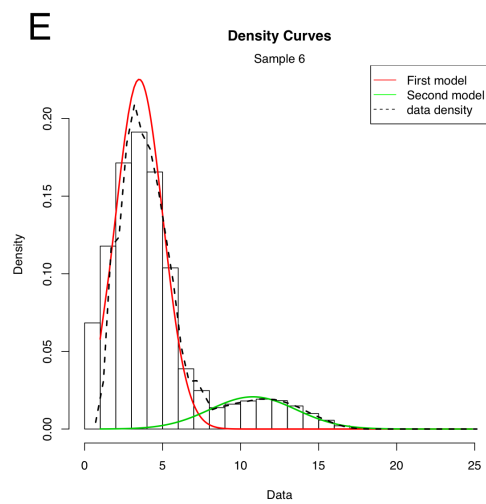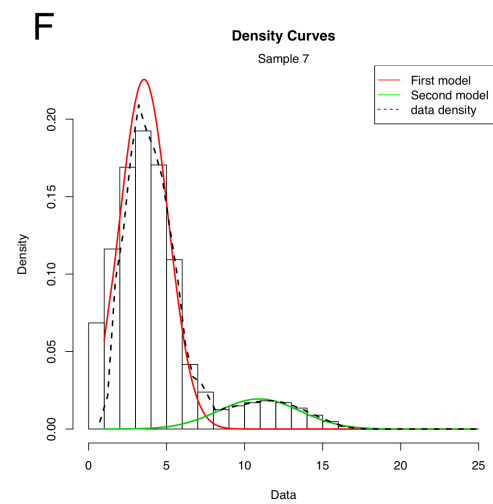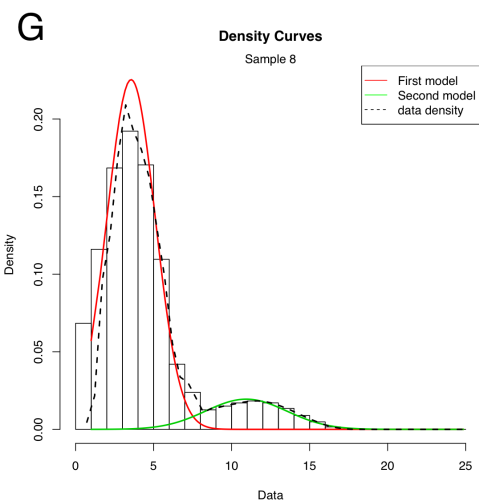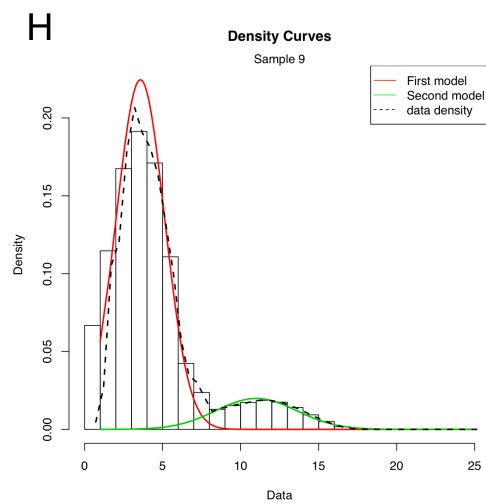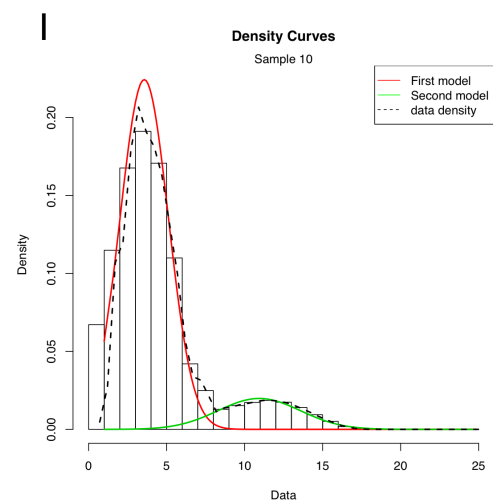

Supplement: Additional file 1 — Figure S1. Histogram of the log2 distance of the nearest CpGs in Sample 1. A spike at zero log2 base pairs distance represents the reverse complement of CpGs (GpC) on the other strand. Figure S2. Consistent distribution shapes across samples. Samples 2-10 are shown from different sequencing depths and samples. (A-I) Red line: First model for regional CpGs; green line: fitted second model for boundary CpGs; Dashed line: density plot of the log2 distances of the nearest CpGs. [file 1471-2105-14-S5-S10-S1.PDF]
